# Exosomal miR‐3131 derived from endothelial cells with KRAS mutation promotes EndMT by targeting PICK1 in brain arteriovenous malformations

**DOI:** 10.1111/cns.14103

**Published:** 2023-01-31

**Authors:** Qiheng He, Ran Huo, Jie Wang, Hongyuan Xu, Shaozhi Zhao, Junze Zhang, Yingfan Sun, Yuming Jiao, Jiancong Weng, Jizong Zhao, Yong Cao

**Affiliations:** ^1^ Department of Neurosurgery, Beijing Tiantan Hospital Capital Medical University Beijing China; ^2^ China National Clinical Research Center for Neurological Diseases Beijing China; ^3^ Beijing Institute of Brain Disorders Beijing China

**Keywords:** brain arteriovenous malformations, endothelial–mesenchymal transition, Kras, microenvironment, miR‐3131, PICK1

## Abstract

**Aims:**

To explore the underlying mechanism by which low‐frequency KRAS mutations result in extensive EndMT occurrence.

**Methods:**

Exosomes derived from primarily cultured brain arteriovenous malformation (bAVMs) and human umbilical vein endothelial cells (HUVECs) transfected with KRAS^G12D^, KRAS^WT^, or KRAS^NC^ lentiviruses were isolated, and their effects on HUVECs were identified by western blotting and immunofluorescence staining. The expression levels of exosomal microRNAs (miRNAs) were evaluated by miRNA microarray, followed by functional experiments on miR‐3131 and detection of its downstream target, and miR‐3131 inhibitor in reversing the EndMT process induced by KRAS^G12D^‐transfected HUVECs and bAVM endothelial cells (ECs) were explored.

**Results:**

Exosomes derived from KRAS^G12D^ bAVM ECs and KRAS^G12D^‐transfected HUVECs promoted EndMT in HUVECs. MiR‐3131 levels were highest in the exosomes of KRAS^G12D^‐transfected HUVECs, and HUVECs transfected with the miR‐3131 mimic acquired mesenchymal phenotypes. RNA‐seq and dual‐luciferase reporter assays revealed that PICK1 is the direct downstream target of miR‐3131. Exosomal miR‐3131 was highly expressed in KRAS^G12D^ bAVM^exos^ compared with non‐KRAS‐mutant bAVM^exos^ or HUVEC^exos^. Finally, a miR‐3131 inhibitor reversed EndMT in HUVECs treated with exosomes or the supernatant of KRAS^G12D^‐transfected HUVECs and KRAS^G12D^ bAVM ECs.

**Conclusion:**

Exosomal miR‐3131 promotes EndMT in KRAS‐mutant bAVMs, and miR‐3131 might be a potential biomarker and therapeutic target in KRAS^G12D^‐mutant bAVMs.

## Graphical abstract

To explore the underlying mechanism by which low‐frequency KRAS mutations result in extensive EndMT occurrence, exosomes were derived from primarily cultured bAVM and HUVECs transfected with KRAS^G12D^, KRAS^WT^, or KRAS^NC^ lentiviruses, and their effects on HUVECs were identified by western blotting and immunofluorescence staining. MiR‐3131 levels were highest in exosomes of KRAS^G12D^‐transfected HUVECs, and HUVECs transfected with miR‐3131 mimic acquired mesenchymal phenotypes. RNA‐seq and dual‐luciferase reporter assays revealed that PICK1 is the direct downstream target of miR‐3131. Exosomal miR‐3131 was highly expressed in KRAS^G12D^ bAVM^exos^ compared with non‐KRAS‐mutant bAVM^exos^ or HUVEC^exos^. Finally, miR‐3131 inhibitor reversed the EndMT in HUVECs treated with exosomes or supernatant of KRAS^G12D^‐transfected HUVECs and KRAS^G12D^ bAVM ECs.

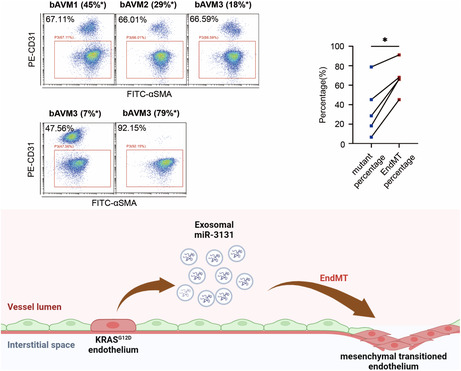

## 1 INTRODUCTION

Brain arteriovenous malformations (bAVMs) are abnormal enlargements of vessels, wherein arterial blood flows directly into draining veins without the normal interposed capillary beds, while no brain parenchyma is contained within the nidus.^1^ bAVMs usually occur in young people, presenting as seizures, neurological deficits, headaches, or devastating spontaneous intracranial hemorrhage.^2^, ^3^ Recently, low‐frequency somatic KRAS mutations were detected in human sporadic bAVMs,^4^, ^5^, ^6^, ^7^, ^8^ and in vivo experiments have confirmed that KRAS mutation can drive abnormal vascular morphology and arteriovenous malformation (AVM) development in mouse and zebrafish models.^9^, ^10^ Previous studies have found that somatic *KRAS* mutations also independently regulate endothelial–mesenchymal transition (EndMT) features.^11^, ^12^

EndMT is a process by which endothelial cells (ECs) can acquire a mesenchymal phenotype.^13^ During EndMT, some EC markers can be lost or retained, while mesenchymal markers and morphology are acquired.^14^, ^15^, ^16^ As EndMT contributes to a multitude of diseases, pharmacological modulation of the signaling pathways underlying EndMT may be effective as a therapeutic treatment.^17^, ^18^

A previous study reported that KRAS mutations occur in bAVM ECs with a low frequency ranging from 2.13% to 52.15%,^7^ while EndMT ECs are widespread in bAVMs.^11^, ^12^, ^19^ Hao et al.^20^ reported that during passaging, cultured bAVM ECs rapidly transition from a cobblestone morphology to a spindle‐shaped morphology. This EndMT phenomenon in bAVMs can be manifested as only a decrease in endothelial markers or an increase in mesenchymal markers(ref). How low‐frequency KRAS mutations result in widespread EndMT occurrence among bAVM ECs remains unclear.

In this study, we utilized bAVM ECs with the KRAS^G12D^ mutation and KRAS^G12D^‐transfected human umbilical vein ECs (HUVECs) to explore the mechanism by which EC–EC communication induces EndMT in bAVMs. Furthermore, we explored the efficacy of a miR‐3131 inhibitor in reversing this EndMT process. The investigation of the underlying mechanism of the process will not only deepen the understanding of the pathogenesis of bAVMs but also contribute to the development of effective medical therapies for bAVMs.

## 2 MATERIALS AND METHODS

### 2.1 Culture and treatment of HUVECs

Commercially available HUVECs (#8000, ScienCell) were cultured in EC medium (ECM, #1001, ScienCell) according to the manufacturer's guidelines and were not used beyond passage 10. The HUVEC lines were cultured and maintained in a humidified atmosphere at 37°C in 5% CO_2_. KRAS^G12D^, KRAS wild type (KRAS^WT^), and KRAS negative control (KRAS^NC^) lentiviruses were used to transfect HUVECs for 24 h.

### 2.2 Cell culture of bAVM ECs

Surgical specimens of bAVMs were obtained from patients at the Department of Neurosurgery, Beijing Tiantan Hospital, Capital Medical University (Beijing, China). Informed consent was obtained to bank bAVM tissues for research purposes. The collection of bAVM tissues for research purposes was approved by the Ethics Committee of Beijing Tiantan Hospital. The bAVM tissues were sealed in sterile glass bottles filled with saline and transferred to the laboratory for further processing.

bAVM ECs were isolated and cultured according to a previous method.^11^ Briefly, the tissue was washed with Dulbecco's phosphate‐buffered saline (DPBS, #D8537, Sigma), cut into small cubes and incubated with 0.1% collagenase I (#C2674, Sigma) at 37°C for 15 min. The predigested tissue was filtered through a 100‐μm cell strainer (#352360, BD Biosciences, USA). The cell suspension was centrifuged at a speed of 300× *g* for 3 min, and the cells were resuspended in ECM. bAVM ECs were isolated using anti‐CD31 Dynabeads (#130–091‐935, Miltenyi Biotec) according to the manufacturer's recommendations. The bAVM ECs were resuspended in ECM in T75 flasks (#3290, Corning).

### 2.3 Whole‐exome sequencing (WES) and droplet digital PCR (ddPCR) validation of bAVM ECs

DNA extraction and whole‐exome sequencing (WES) of primary cultured bAVM ECs were performed according to previous methods.^11^ Briefly, commercially available kits (Gentra Puregene, QIAGEN) were used to isolate genomic DNA from bAVM ECs. We used two methods to examine the quality of DNA: DNA degradation and contamination were monitored on 1% agarose gels, and the concentration was measured with a Qubit® 2.0 Fluorometer (Invitrogen). We used a total of 0.6 μg of genomic DNA per case for library preparation. Sequencing libraries were generated, and index codes were added with an Agilent SureSelect Human All Exon V6 kit (Agilent Technologies) according to the manufacturer's recommendations. The index‐coded samples were clustered on a cBot Cluster Generation System using a HiSeq PE Cluster Kit (Illumina). After cluster generation, the DNA libraries were sequenced on an Illumina HiSeq platform, and 150 bp paired‐end reads were generated. The KRAS^G12D^ mutation was further validated using droplet digital polymerase chain reaction (ddPCR). Ultimately, 6 out of 20 bAVM patients were found to have the KRAS^G12D^ mutation in their ECs.

### 2.4 Flow cytometry validation of EndMT in ECs

To assess the EndMT rate in bAVM ECs with KRAS mutation, we examined the typical EndMT markers CD31 and α‐SMA in bAVM ECs using flow cytometry. Briefly, cell samples were collected using a standard method and incubated with an antibody against the cell surface protein CD31 (#303106, BioLegend) in cell staining buffer (#420201, BioLegend) for 15 min at room temperature in the dark. Next, the cells were washed with DPBS three times and fixed using fixation buffer (#420801, BioLegend) for 40 min at 4°C. Then, the cells were washed with permeabilization wash buffer (#421002, BioLegend) three times and stained with an antibody against intracellular α‐SMA (#197240, Abcam) at 4°C overnight. We used unstained cells as controls. With regard to gating, forward scatter (FSC) versus side scatter (SSC) was used to eliminate debris. In the following experiments, CD31 + αSMA‐ cells were recognized as normal ECs, whereas CD31‐α‐SMA ± cells were recognized as EndMT cells.

### 2.5 Isolation of exosomes from culture medium

KRAS^G12D^‐mutant bAVM ECs, KRAS^WT^ bAVM ECs, or HUVECs were cultured in exosome‐depleted ECM for 48 h, and the culture supernatant was collected to isolate exosomes. To prevent contamination and interference of the transfected lentiviruses, the transfected HUVECs were washed three times with DPBS every passage and cultured for more than 7 days before exosomes were extracted as described in previous studies. Exosomes were isolated using differential ultracentrifugation as previously reported.^21^ Briefly, the culture supernatant was centrifuged twice at 3000× *g* for 15 min at 4°C and then at 10,000× *g* for 30 min to remove cells and debris. The supernatant was further filtered with a 0.22‐μm filter (Millipore). The obtained medium was centrifuged at 110,000× *g* for 90 min at 4°C to pellet exosomes. The supernatant was discarded and finally resuspended again in PBS. We further confirmed the lack of eGFP expression in HUVECs cocultured with exosomes to verify that there was no lentivirus contamination.

### 2.6 Identification of exosomes

The morphology of exosomes was observed by transmission electron microscopy (TEM, Tecnai G2 Spirit BioTWIN, FEI). Briefly, exosomes were fixed with 4% paraformaldehyde and spotted onto glow‐discharged copper grids. The copper grids were dried for 5 min at room temperature. The samples were stained with 1% uranyl acetate for 1 min. Then, the samples were dried and observed at 80 kV. The size distribution of exosomes was observed using nanoparticle tracking analysis (NTA; ZetaView S/N 17–310) and analyzed using OriginPro 8.5 (OriginLab). Western blot analysis was performed to detect the exosome markers CD63, CD81, and TSG101.

### 2.7 miRNA mimic, miRNA inhibitor, and siRNA transfection

ECs were transfected with a miR‐3131 mimic, a negative control mimic (NC mimic), a miR‐3131 inhibitor, an NC inhibitor, PICK1‐targeting siRNA (siPICK1), or negative control siRNA (siNC) using Lipofectamine 3000 (Invitrogen). After transfection for 48 h, the cells were harvested for subsequent experiments. The synthesized hsa‐miR‐3131 mimic sequence was UCGAGGACUGGUGGAAGGGCCUU, and the synthesized hsa‐miR‐3131 inhibitor sequence was AAGGCCCUUCCACCAGUCCUCGA (SyngenTech).

### 2.8 RNA sequencing (RNA‐seq)

The TRIzol method was used to prepare samples for RNA‐seq. RNA purity and concentration were measured using a Qubit® 2.0 Fluorometer (Life Technologies). An RNA Nano 6000 Assay Kit and a Bioanalyzer 2100 system (Agilent Technologies) were utilized to assess RNA integrity. RNA‐seq was performed according to a previous method.^22^

### 2.9 Dual‐luciferase reporter gene assay

For luciferase reporter assays, the putative miR‐3131 complementary site in the 3′ untranslated region (3′UTR) segment of the PICK1 gene was amplified by PCR and inserted into vectors (SyngenTech). Site‐directed mutagenesis of the miR‐3131 target site in the PICK1 3′UTR was performed using a QuikChange Site‐Directed Mutagenesis Kit (Agilent Technologies). The PICK1 3'UTR‐WT or PICK1 3′UTR‐MUT plasmid (SyngenTech) was cotransfected with a miR‐3131 lentiviral vector into the indicated cells by using Lipofectamine 3000 (Invitrogen). Luciferase activity was measured 48 h after transfection with a Dual‐luciferase Reporter Assay System (Promega). The Renilla luciferase signal was used for normalization. Each assay was repeated in three independent experiments.

### 2.10 Statistical analysis

SPSS version 25.0, GraphPad Prism version 8.00, and R version 4.0.5 were used for statistical analyses. The K–S test for normality was used to assess data distribution. For normally distributed data, *t*‐tests or one‐way ANOVA were used to assess differences between two groups or among three groups of quantitative variables. For data not normally distributed, nonparametric tests were used. A two‐tailed probability value of 0.05 or less was considered to indicate statistical significance.

## 3 RESULTS

### 3.1 The percentage of EndMT ECs in bAVMs is markedly greater than the percentage of ECs with KRAS mutation

**FIGURE 1:**
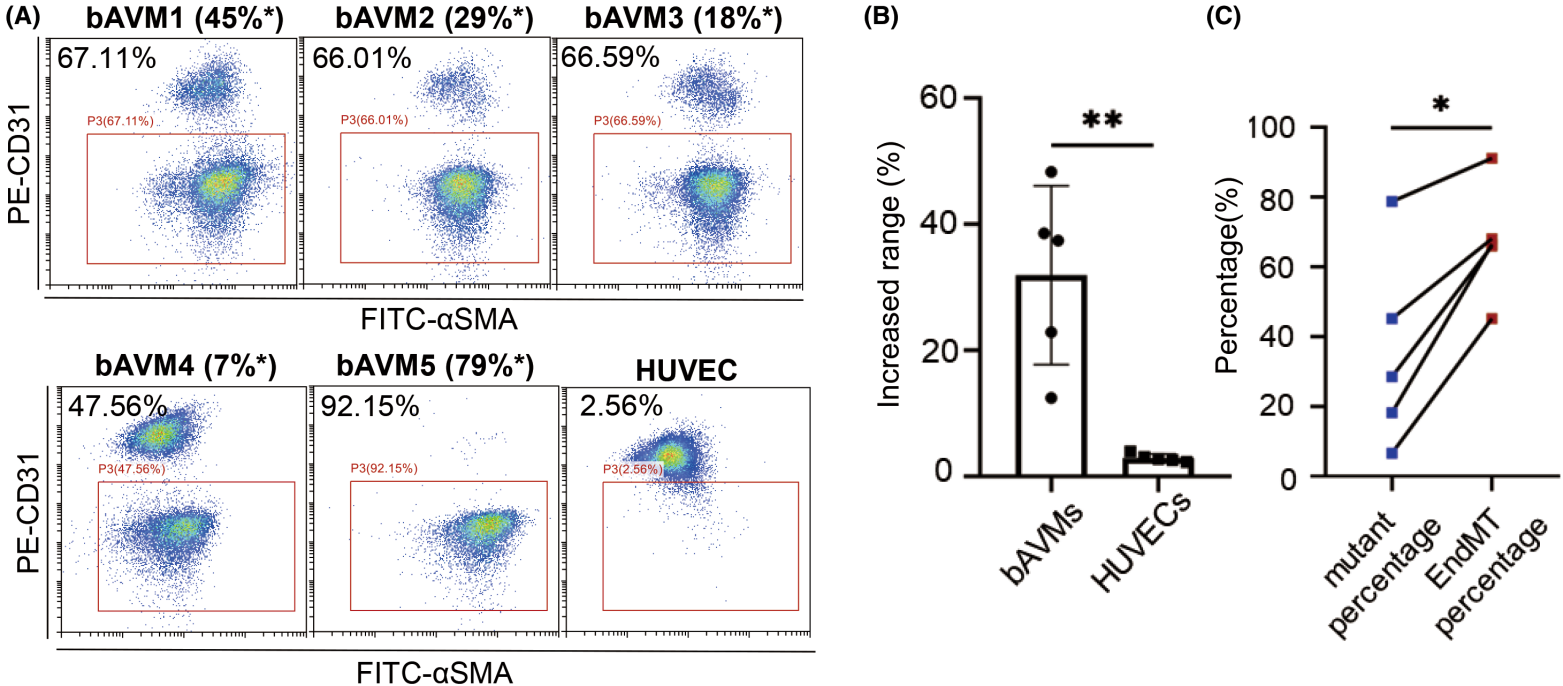
The percentage of EndMT ECs in bAVMs was markedly greater than the percentage of ECs with KRAS mutation. (A) Flow cytometric analysis of the expression of CD31 and αSMA and the percentage of KRAS^G12D^‐mutant ECs in bAVM ECs (*n* = 5) and representative HUVECs are shown. (B) Scatter plot showing the mutant percentage and EndMT percentage in bAVM primary ECs (*n* = 5). *p < 0.05 (C) Bar plot with scattering showing the increased percentage of EndMT cells compared to mutant cells in bAVMs (*n* = 5) and HUVECs (*n* = 5). EndMT, endothelial–mesenchymal transition; EC, endothelial cell; bAVM, brain arteriovenous malformation. *The percentage of KRAS^G12D^‐mutant ECs in bAVM ECs is shown in brackets.

To compare the percentage of EndMT ECs with the percentage of ECs with KRAS mutation in bAVMs, we simultaneously performed WES and flow cytometry analysis on primary bAVM ECs (*n* = 5) or HUVECs (*n* = 5). The allele frequency of KRAS^G12D^ in bAVM ECs as determined by WES and confirmed by ddPCR is shown in Table S1. The percentage of ECs with KRAS mutation was calculated according to a twofold allele frequency in WES. The corresponding EndMT percentage was obtained by flow cytometry (Figure 1A, Figure S1A). We found that the percentage of EndMT ECs was significantly higher than the percentage of ECs with KRAS mutation in the bAVMs, as shown in Figure 1B (paired *t* test, *p* = 0.010). Furthermore, we found only a small proportion of EndMT in normal ECs, and EndMT increased significantly in KRAS^G12D^ bAVMs compared to normal ECs (*p* = 0.0079, Figure 1C). Our results support the idea that KRAS^G12D^ may play a role in the EndMT phenotype transmitted to WT cells.

### 3.2 Exosomes derived from bAVM ECs with *KRAS* ^G12D^ mutation promote EndMT in HUVECs

**FIGURE 2:**
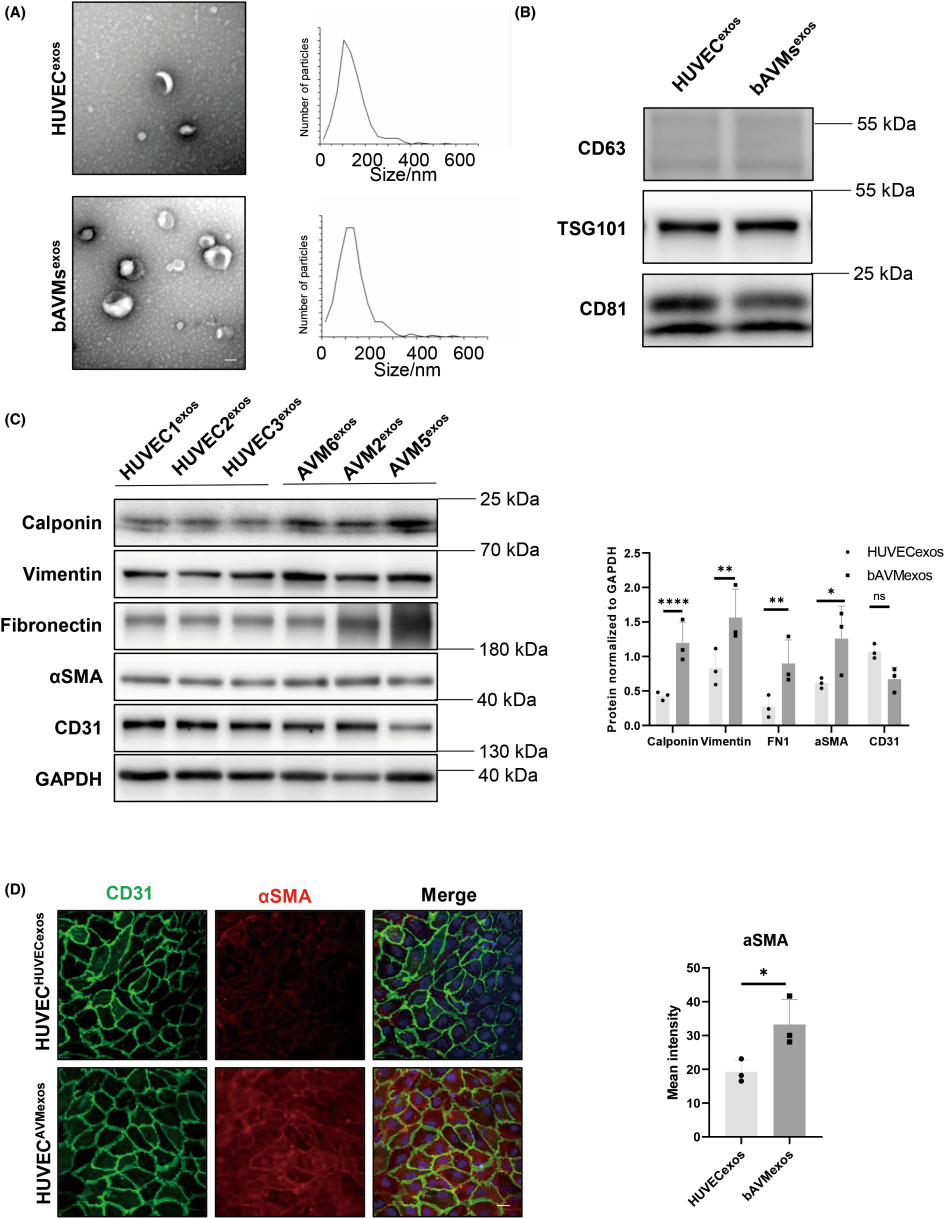
Exosomes derived from bAVM ECs with KRAS^G12D^ mutations promote EndMT in HUVECs. (A) TEM and NTA of bAVM‐derived exosomes (bAVM^exos^) and HUVEC‐derived exosomes (HUVEC^exos^) showed 50–150 nm cup‐shaped particles (*n* = 3). One representative image of three is shown. (B) The protein levels of the exosomal markers CD63, CD81, and TSG101 were detected by western blot assay in bAVM^exos^ and HUVEC^exos^ (*n* = 3). One representative image of three is shown. (C) Western blot assay results showing the effects of bAVM^exos^ and HUVEC^exos^ on calponin, vimentin, fibronectin, αSMA, and CD31 in HUVECs (*p* < 0.05, *n* = 3). (D) Immunofluorescence staining for CD31 and αSMA in HUVECs subjected to different treatments (HUVEC^exos^ or bAVM^exos^). One representative image of three is shown. bAVM, brain arteriovenous malformation; EC, endothelial cell; EndMT, endothelial–mesenchymal transition; HUVEC, human umbilical vein endothelial cell; TEM, transmission electron microscopy; NTA, nanoparticle tracking analysis.

Previous studies have suggested that exosomes play a vital role in communication between cells. For example, in studies on the tumor microenvironment, exosomes secreted from KRAS‐mutant prostate cancer cell lines have been found to contain small RNAs and to induce aggressive tumors in secondary recipients.^23^ Therefore, we hypothesized that bAVM ECs with *KRAS* ^G12D^ mutations might secrete exosomes to promote EndMT in neighboring *KRAS* ^wt^ ECs. To test this hypothesis, exosomes were extracted from conditioned media of bAVM ECs with *KRAS* ^G12D^ mutation using differential ultracentrifugation. For identification of exosomes in bAVM samples (2, 5, and 6), we used TEM and NTA to determine that the exosomes had a characteristic cup‐shaped appearance and a mean size of 50–150 nm (Figure 2A). Additionally, the specific expression of the typical exosomal marker proteins CD63, CD81, and TSG101 was identified using western blot assay (Figure 2B).

Then, in vitro experiments were performed to evaluate the effect of exosomes secreted by primary ECs derived from *KRAS* ^G12D^ bAVM ECs, and exosomes of normal HUVECs were used as control exosomes. As EndMT is widespread in bAVMs,^11^, ^12^ and the activation of the MAPK–ERK pathway occurs in ECs of the samples obtained from patients regardless of the presence of a KRAS mutation,^7^ it is not assured that exosomes secreted by non‐KRAS‐mutant bAVM ECs have the ability to induce EndMT in HUVECs. Therefore, exosomes from nonmutant bAVM ECs were not used as controls. After coculture with exosomes from primary bAVM ECs for 3 days, the protein levels of the mesenchymal markers calponin, vimentin, fibronectin, and α‐SMA were all significantly increased (*p* < 0.05, Figure 2C). Immunofluorescence staining also showed an increased level of α‐SMA in HUVECs treated with bAVM^exos^ derived from bAVM2 (*p* < 0.05, Figure 2D). These results revealed that bAVM ECs with the *KRAS* ^G12D^ mutation were able to secrete exosomes to promote EndMT in HUVECs.

### 3.3 Exosomes derived from KRAS^G12D^ ‐transfected HUVECs promote EndMT in HUVECs

**FIGURE 3:**
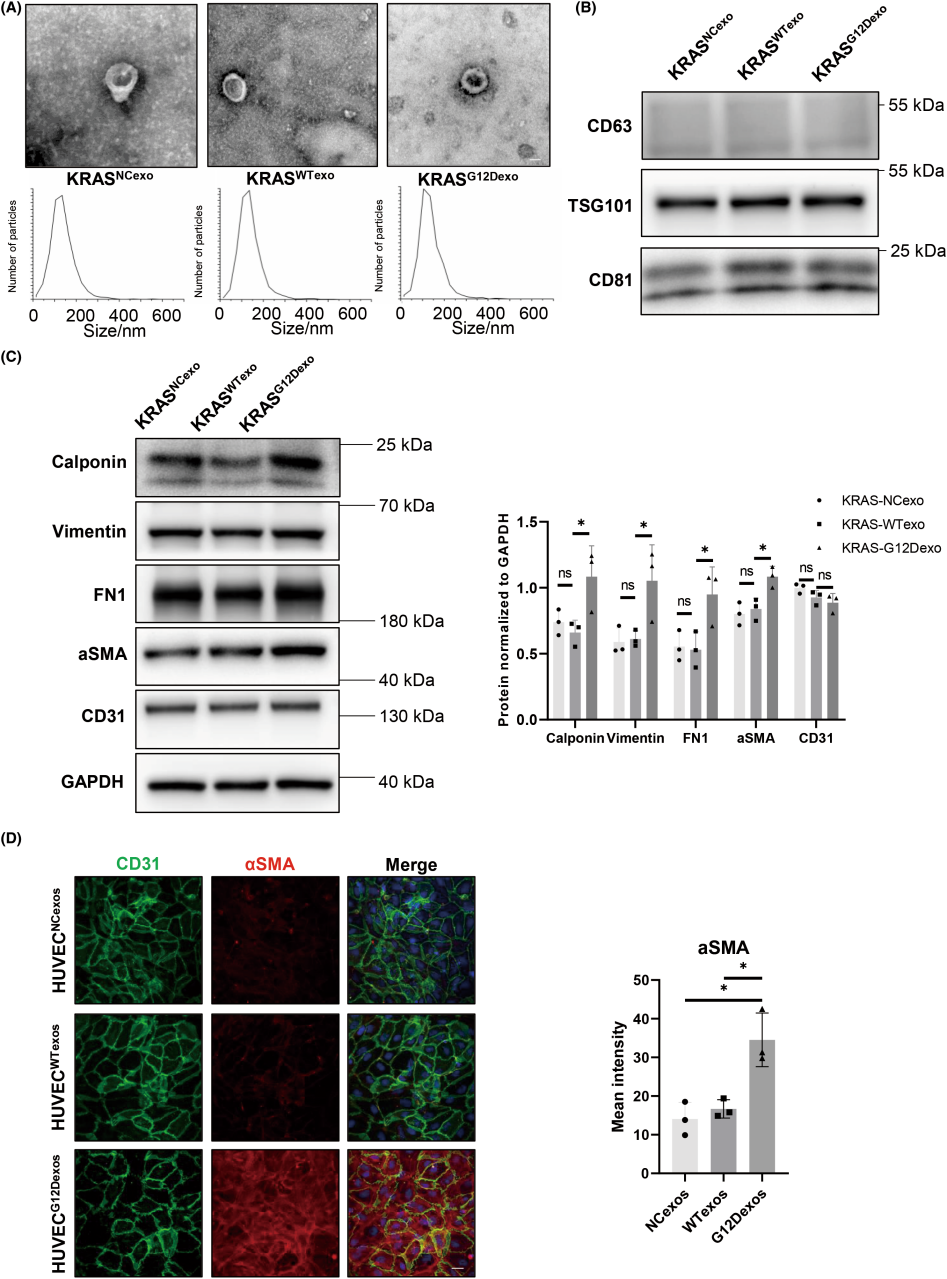
Exosomes derived from HUVECs transfected with KRAS^G12D^ promote EndMT in HUVECs. (A) TEM and NTA of exosomes derived from HUVECs transfected with KRAS^G12D^, KRAS^WT^, or KRAS^NC^ lentiviruses (HUVEC^G12Dexos^, HUVEC^WTexos^, or HUVEC^NCexos^) (*n* = 3). One representative image of three is shown. (B) Protein expression of the exosomal markers CD63, CD81, and TSG101 in a western blot assay (*n* = 3). (C) Western blot assay results showing the effect of HUVEC^G12Dexos^, HUVEC^WTexos^, or HUVEC^NCexos^ on calponin, vimentin, fibronectin, αSMA, and CD31 in HUVECs (*p* < 0.05, *n* = 3). (D) Immunofluorescence staining for CD31 and αSMA in HUVECs subjected to different treatments (HUVEC^G12Dexos^, HUVEC^WTexos^, or HUVEC^NCexos^). One representative image of three is shown. HUVECs, human umbilical vein endothelial cells; EndMT, endothelial–mesenchymal transition; TEM, transmission electron microscopy; NTA, nanoparticle tracking analysis.

To further confirm the mediating effect of exosomes from KRAS^G12D^‐mutant ECs on EndMT, HUVECs were transfected with KRAS^G12D^, KRAS^WT^, or KRAS^NC^ lentiviruses. Then, exosomes were extracted and identified using differential ultracentrifugation. TEM and NTA identified 50–150 nm cup‐shaped extracellular vesicles, and the typical markers CD63, CD81, and TSG101 were highly expressed in these exosomes (Figure 3A,B).

Furthermore, the function of exosomes derived from KRAS^G12D^‐mutant HUVECs was studied. After coculture with exosomes secreted by *KRAS* ^G12D^ ECs, western blotting showed increased protein levels of the mesenchymal markers calponin, vimentin, fibronectin, and α‐SMA compared with HUVECs cocultured with exosomes secreted by *KRAS* ^WT^ or *KRAS* ^NC^ ECs (*p* < 0.05, Figure 3C, Figure S1A). The mean intensity of α‐SMA protein in the immunofluorescence staining assay also increased after coculture with *KRAS* ^G12D^ EC‐secreted exosomes (*p* < 0.05, Figure 3D). These results revealed that exosomes secreted from *KRAS* ^G12D^ ECs promoted the EndMT process in HUVECs.

### 3.4 Significant amounts of miR‐3131 are contained in exosomes derived from HUVECs transfected with KRAS^G12D^ , and exosomal miR‐3131 can promote EndMT in HUVECs

**FIGURE 4:**
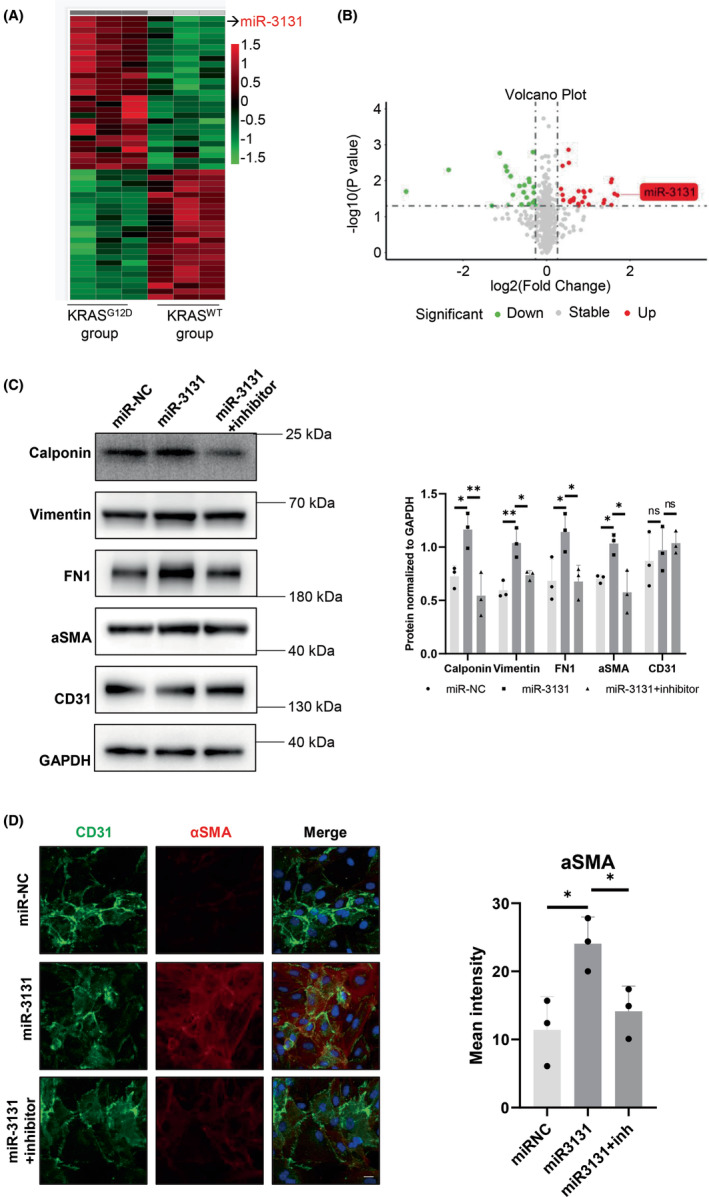
miR‐3131 is significantly highly expressed in exosomes derived from HUVECs transfected with KRAS^G12D^ and can promote EndMT in HUVECs. (A) Heatmap of differentially expressed miRNAs in exosomes derived from HUVECs transfected with KRAS^G12D^ or KRAS^WT^ lentiviruses. In total, 50 were upregulated and 101 were downregulated. A fold change≥1.2 and a *p* value < 0.05 were considered to indicate differential regulation. (B) Volcano plot of differentially expressed miRNAs in HUVEC^G12Dexos^ and HUVEC^WTexos^. miR‐3131 is highlighted. (C) Effects of miR‐3131 mimic and miR‐3131 inhibitor on the expression of calponin, vimentin, fibronectin, αSMA, and CD31 in HUVECs as determined by western blot assays (*p* < 0.05, *n* = 3). (D) Immunofluorescence staining for CD31 and αSMA in HUVECs subjected to miR‐3131 mimic and miR‐3131 inhibitor treatment. One representative image of three is shown. HUVEC, human umbilical vein endothelial cell; EndMT, endothelial–mesenchymal transition.

Exosomal microRNAs (miRNAs) are some of the most important molecules in cell–cell communication.^24^, ^25^, ^26^, ^27^, ^28^ To identify the responsible exosomal miRNAs in the current study, differentially expressed miRNAs were evaluated by miRNA microarray assay, and exosomes from *KRAS* ^G12D^ ECs, *KRAS* ^WT^ ECs, and *KRAS* ^NC^ ECs were sequenced. Finally, 50 and 101 exosomal miRNAs were identified as differentially expressed miRNAs in *KRAS* ^G12D^ versus *KRAS* ^WT^ ECs and in *KRAS* ^G12D^ versus *KRAS* ^NC^ ECs according to the thresholds of *p* < 0.05 and a fold change >1.2. Specifically, 27 were upregulated and 23 were downregulated in *KRAS* ^G12D^ versus *KRAS* ^WT^ ECs (Figure 4A,B), while 55 were upregulated and 46 were downregulated in *KRAS* ^G12D^ versus *KRAS* ^NC^ ECs (data for *KRAS* ^G12D^ EC exosomes versus *KRAS* ^NC^ EC exosomes not shown). Among all differentially expressed miRNAs, miR‐3131 was the most highly expressed miRNA in *KRAS* ^G12D^ ECs compared with *KRAS* ^WT^ ECs (fold change = 3.24, *p = 0.025), as shown in the volcano plot, and miR‐3131 was also significantly upregulated in *KRAS* ^G12D^ ECs compared with *KRAS* ^NC^ ECs (fold change = 15.83, *p* = 0.005). The results were further validated by reverse transcription–PCR (RT–PCR), which showed that exosomal miR‐3131 levels were significantly elevated in *KRAS* ^G12D^ ECs compared with *KRAS* ^WT^ ECs (fold change = 4.03, *p* = 0.020) and in *KRAS* ^G12D^ ECs compared with *KRAS* ^NC^ ECs (fold change = 453.66, **p = 0.0024, Figure S1A). We also tested miR‐3131 in *KRAS* ^G12D^ ECs and found that the endogenous miR‐3131 level was also significantly higher in KRAS^G12D^ ECs than in KRAS^WT^ ECs (fold change = 1.40, *p = 0.0266) and KRAS^NC^ ECs (fold change = 1.39, *p = 0.0255) by RT–PCR (Figure S1A).

To further investigate the biological function of miR‐3131 in the EndMT process, HUVECs were transfected with miR‐3131 mimic, and western blotting was used to determine the protein expression level. After transfection with miR‐3131, the protein levels of calponin, vimentin, fibronectin, and α‐SMA were significantly increased (*p* < 0.05), and the miR‐3131 inhibitor reversed this process (*p* < 0.05, Figure 4C, Figure S1A). The level of α‐SMA protein was significantly increased after transfection with the miR‐3131 mimic (*p* < 0.05), and this effect was reversed by the miR‐3131 inhibitor, as identified using an immunofluorescence staining assay (*p* < 0.05, Figure 4D). These data demonstrated that miR‐3131 in exosomes from KRAS^G12D^‐mutant HUVECs could promote EndMT in HUVECs.

### 3.5 PICK1 is the downstream target of miR‐3131 in EndMT regulation

**FIGURE 5:**
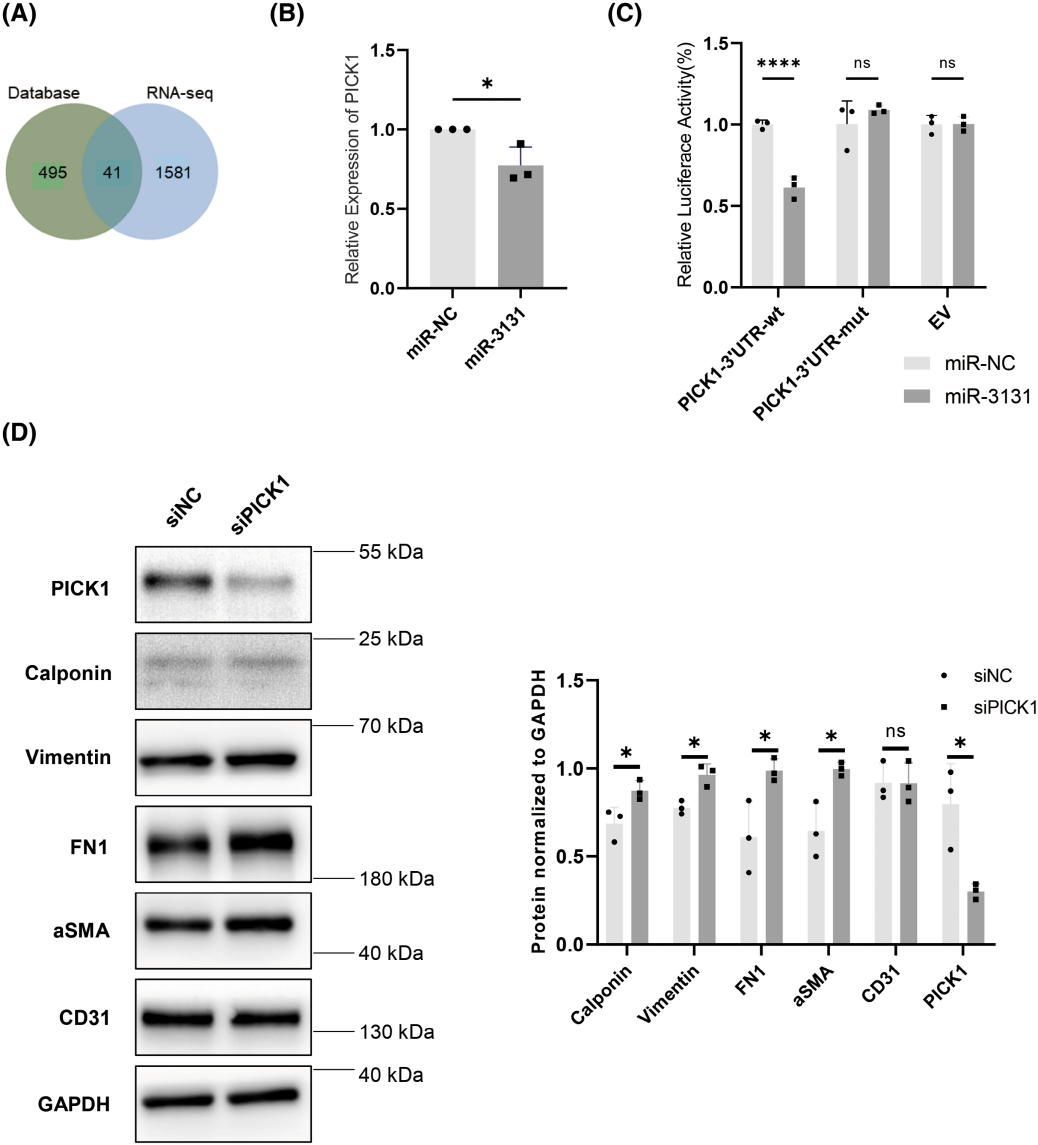
PICK1 is the downstream target of miR‐3131 in EndMT regulation. (A) Venn diagram of the results obtained from four databases (miRanda, RNA22, RNAhybrid, and TargetScan) and RNA‐seq. (B) RT–PCR results showing decreased expression of PICK1 in miR‐3131‐transfected HUVECs. *p < 0.05. (C) Scatter plot of the effects of miR‐NC and miR‐3131 expression on the luciferase activity of the PICK1 3'UTR wild type, 3'UTR mutant, and control groups. ****p < 0.0001. (D) Expression of PICK1 in HUVECs transfected with siPICK1 and the expression of calponin, vimentin, fibronectin, αSMA, and CD31 (*p* < 0.05, *n* = 3). **p < 0.01, ****p < 0.0001. EndMT, endothelial–mesenchymal transition; HUVEC, human umbilical vein endothelial cell.

To further determine the downstream target of miR‐3131, four databases (miRanda, RNA22, RNAhybrid, and TargetScan) were utilized to predict the potential downstream targets of miR‐3131, and RNA‐seq was used to assess the mRNA changes in HUVECs after transfection with the miR‐3131 mimic. A total of 536 potential genes were predicted to be direct downstream targets of miR‐3131 in all four databases, while 1622 genes were significantly downregulated in the RNA‐seq expression profile of miR‐3131 mimic‐transfected HUVECs compared with NC mimic‐transfected HUVECs (fold change > 1.2, *p* < 0.05, Figure 5A). Among them, 41 genes were identified as overlapping genes, and PICK1 has been reported to be a negative regulator of the TGF‐β pathway, which plays a vital role in EndMT by promoting caveolin‐dependent degradation of the TGF‐β type I receptor.^29^, ^30^, ^31^ To confirm the mRNA level of PICK1 in miR‐3131‐transfected HUVECs, RT–PCR was used and identified significantly lower mRNA levels of PICK1 in miR‐3131 mimic‐transfected HUVECs (fold change = 0.77, *p* = 0.028, Figure 5B). To substantiate the site‐specific repression of PICK1 by miR‐3131, we constructed a mutated PICK1 3′ UTR luciferase reporter. The dual‐luciferase reporter assay showed that the luciferase activity of PICK1 with the WT 3'UTR was significantly suppressed with an efficiency of 39% in miR‐3131‐expressing HEK 293FT cells (*p* < 0.05). In contrast, the luciferase activity of PICK1 with the 3′UTR mutation was not changed (Figure 5C). These results indicate that PICK1 is the downstream target of miR‐3131.

To further investigate the function of PICK1 in ECs, we knocked down PICK1 in HUVECs using siRNA. After transfection with PICK1 siRNA, the protein levels of the mesenchymal markers calponin, vimentin, fibronectin, and α‐SMA were all increased in PICK1‐knockdown HUVECs (*p* < 0.05, Figure 5D, Figure S1A). These findings indicated that PICK1 was the direct downstream target of miR‐3131, and that the knockdown of PICK1 promoted EndMT in HUVECs.

### 3.6 miR‐3131 levels are significantly increased in exosomes from *KRAS* ^G12D^ bAVM ECs, and miR‐3131 inhibitor treatment can reverse EndMT in HUVECs treated with exosomes or supernatant from KRAS^G12D^ bAVM ECs

**FIGURE 6:**
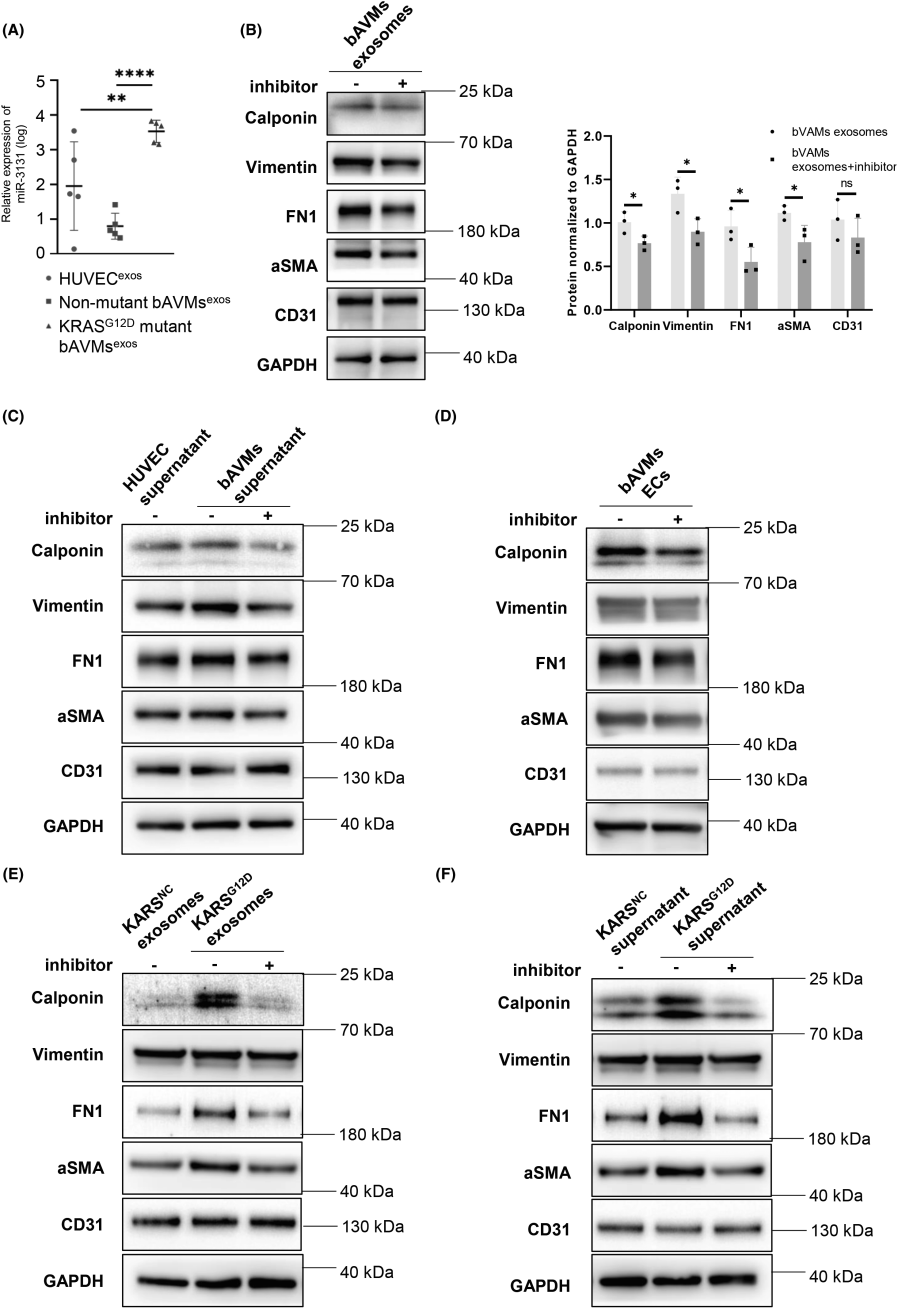
miR‐3131 expression is significantly increased in exosomes from bAVM ECs with *KRAS* ^G12D^ mutation, and miR‐3131 inhibitor treatment can reverse EndMT in HUVECs treated with exosomes or supernatant from KRAS^G12D^ bAVM ECs. (A) Scatter plot of the relative expression level of miR‐3131 in exosomes derived from HUVECs and bAVM ECs. (B–F) Western blot assay results for HUVECs subjected to different treatments: (B–C) Effects of a miR‐3131 inhibitor on HUVECs treated with exosomes or supernatant derived from bAVM ECs. (*p* < 0.05, *n* = 3) (D) Effect of a miR‐3131 inhibitor on bAVM ECs (patient 2). (E–F) Effect of a miR‐3131 inhibitor on HUVECs treated with exosomes or supernatant derived from KRAS^G12D^‐ or KRAS^NC^‐transfected HUVECs (*p* < 0.05, *n* = 3). bAVM, brain arteriovenous malformation; EC, endothelial cell; EndMT, endothelial–mesenchymal transition; HUVEC, human umbilical vein endothelial cell.

Exosomal miRNAs are often reported as potential biomarkers in vascular diseases.^32^, ^33^ To identify the clinical value of exosomal miR‐3131 in bAVMs, exosomes were isolated from the ECs of *KRAS* ^G12D^ bAVMs (*n* = 5), *KRAS* ^wt^ bAVMs (*n* = 5), and HUVECs (*n* = 5). Total exosomal miRNAs were extracted, and RT–PCR revealed that the expression level of miR‐3131 was significantly higher in *KRAS* ^G12D^ bAVM^exos^ than in *KRAS* ^wt^ bAVM^exos^ (fold change = 498.36, *p* < 0.001) and HUVEC^exos^ (fold change = 33.41, *p* = 0.031, Figure 6A). These results imply that exosomal miR‐3131 is a potential biomarker in KRAS^G12D^ bAVM patients.

To further explore the efficacy of the miR‐3131 inhibitor in reversing EndMT in bAVMs, we utilized a miR‐3131 inhibitor in HUVECs treated with the exosomes or supernatant of KRAS^G12D^‐mutant bAVM ECs, and a miR‐3131 inhibitor was also used to treat KRAS^G12D^‐mutant bAVM ECs. After treatment with the miR‐3131 inhibitor, the protein levels of calponin, fibronectin, αSMA, and vimentin were significantly decreased in HUVECs treated with the exosomes or supernatant of KRAS^G12D^‐mutant bAVM ECs (*p* < 0.05, Figure 6B,C; Figures S1). In two of four cases (patient 2, allele frequency 14.29%, and patient 3, allele frequency 9.09%) with available KRAS^G12D^‐mutant bAVM ECs, the protein levels of the mesenchymal markers calponin, fibronectin, αSMA, and vimentin were decreased by the miR‐3131 inhibitor (patient 2, in Figure 6D, and patient 3, Figure S1), while in the other two (patient 4, allele frequency 3.33%, and patient 1, allele frequency 22.62%), there were no changes (Figure S1). Whether the efficacy of the miR‐3131 inhibitor in KRAS^G12D^‐mutant bAVM ECs themselves depends on the allele mutation frequency requires further research.

To validate the effect of the miR‐3131 inhibitor on the communication between KRAS‐mutant ECs and normal ECs, we administered the miR‐3131 inhibitor to HUVECs treated with exosomes or supernatant derived from KRAS^G12D^‐transfected HUVECs. The protein levels of the mesenchymal markers calponin, fibronectin, αSMA, and vimentin were significantly increased in cells treated with exosomes or supernatant from KRAS^G12D^‐transfected HUVECs and were effectively reversed by the miR‐3131 inhibitor (*p* < 0.05, Figure 6 E,F; Figures S1).

## 4 DISCUSSION

In this study, we found that bAVM ECs with *KRAS* ^G12D^ mutations are able to secrete exosomes to promote EndMT in neighboring *KRAS* ^WT^ ECs. Furthermore, we found that exosomal miR‐3131 derived from ECs with KRAS mutations exerts this effect by targeting PICK1. Our results imply that miR‐3131 is a potential biomarker, and that miR‐3131 inhibitors are promising candidate treatment agents for KRAS‐mutated bAVMs.

Intercellular communication through either cell‐to‐cell contact or paracrine effects is believed to be a key process in vascular remodeling.^34^, ^35^ Exosomes, which are newly identified natural nanocarriers and intercellular messengers, play a pivotal role in regulating cell‐to‐cell communication and have emerged as important mediators in the pathogenesis of vascular diseases, including atherosclerosis, neointima formation (and vascular repair), primary hypertension, pulmonary artery hypertension, and aortic aneurysm.^33^, ^36^, ^37^, ^38^, ^39^ Exosomes show excellent prognostic and therapeutic potential for vascular diseases. Our study suggests that in addition to somatic KRAS mutations, EC–EC communication also contributes to the pathogenesis and development of bAVMs. Widespread EndMT during the development of bAVMs is caused not only by KRAS mutation^11^ but also by the effects of KRAS‐mutant ECs on nonmutant ECs.

MiRNAs have been observed in secreted exosomes, and many cells can secrete miRNAs via exosomes to exert their regulatory effects on recipient cells.^21^ miRNAs are small single‐stranded noncoding RNA molecules that bind to mRNA, promoting cleavage and subsequent degradation of the mRNA.^40^ The exosomal transfer of miRNAs is a novel mechanism for intercellular communication. Modification of the function of miRNAs to target multiple mRNAs and regulate gene expression is a new therapeutic approach for cancer.^41^ Previous studies have indicated that overexpression of miR‐3131 promotes proliferation and inhibits apoptosis in hepatocellular carcinoma (HCC) cell lines and that miR‐3131 may act as a proto‐oncogene in HCC.^42^ The miR‐3131 downstream target gene PICK1 can regulate the TGF‐β signaling pathway by promoting caveolin‐dependent degradation of the TGF‐beta type I receptor.^29^ Decreased expression of PICK1 regulates the metastasis of cancer cells and is associated with EndMT.^31^ Our study indicates that exosomal miR‐3131 in the microenvironment plays a crucial role in the EndMT process, miR‐3131 is a potential biomarker, and miR‐3131 inhibitors might be promising candidates for the medical treatment of KRAS‐mutated bAVMs. However, further research is needed to determine how KRAS mutation induces ECs to secrete miR‐3131 in exosomes, which are themselves potential targets for bAVM treatment.

## AUTHOR CONTRIBUTIONS

Y.C. designed the study. Q.H., R.H., and H.X. wrote the manuscript and performed the statistical analysis. Q.H., R.H., J.W., and H.X. performed the experiments. S.Z., J.Z., Y.S., Y.J., and J.W. collected data and patient samples. J. Z and Y.C. supervised the study. Y.C. reviewed the manuscript.

## FUNDING INFORMATION

This article was funded by the project “Genomics Platform Construction for Chinese Major Brain Disease‐AVM” (No. PXM2019_026280_000002‐AVM), Beijing Advanced Innovation Center for Big Data‐based Precision Medicine (PXM2020_014226_000066).

## CONFLICT OF INTEREST STATEMENT

None.

## Supporting information


AppendixS1

## Data Availability

The data that support the findings of this study are available from the corresponding author upon reasonable request.
