# Supplementary material for: Exosomal miR‐3131 derived from endothelial cells with KRAS mutation promotes EndMT by targeting PICK1 in brain arteriovenous malformations
Source: CNS Neurosci Ther. 2023 Jan 31;29(5):1312–24. doi: 10.1111/cns.14103 (PMC10068464; doi:10.1111/cns.14103)
Supplement: Supplementary file 1 — AppendixS1 [file CNS-29-1312-s001.pdf]

Supplementary Materials

Appendix S1. Western blot assay

Proteins were extracted with radioimmunoprecipitation assay lysis buffer (Sigma, USA) and quantified with a bicinchoninic acid protein assay kit (KeyGen, China). Equivalent amounts of cell lysates were separated using sodium dodecyl sulfate–polyacrylamide gel electrophoresis and transferred to polyvinylidene fluoride transfer membranes (Millipore, USA). The membranes were blocked for an hour at room temperature, immunoblotted overnight at 4°C with antibodies and then incubated with appropriate secondary antibodies. The bands were visualized using Pierce electrochemiluminescence western blotting substrate (Thermo Fisher Scientific, USA).

Appendix S2. Reverse transcription–polymerase chain reaction (RT–PCR)

Total RNA obtained from cultured cells was extracted using a FastPure Cell/Tissue Total RNA Isolation Kit V2 (RC112, Vazyme, China). cDNA was then reverse-transcribed and amplified by PCR using a PrimeScript™ RT Reagent Kit (RR047, Takara, Japan). RT–PCR was performed using TB Green Premix Ex Taq (RR082, Takara, Japan) with a QuantStudio™ 3 System (Applied Biosystems, Thermo Scientific, USA) with specific primers. Relative quantification was performed using the ΔΔCt method with GAPDH and U6 as the reference genes for mRNA and miRNA, respectively.

Appendix S3. Immunofluorescence staining

Cells were seeded in a μ-Slide 8-well high glass-bottom chamber slide (ibidi, Germany), cultured for 48 h, and then fixed with 4% paraformaldehyde. After

permeabilization in 0.3% Triton X-100 and blocking, the cells were exposed to primary antibodies against CD31 (1:800, #3528, CST, USA) and αSMA (1:200, #19245, CST, USA) at 4°C overnight and to goat anti-mouse IgG-HRP (1:5000, M21001, Abmart, China) or goat anti-rabbit IgG-HRP (1:5000, M21002, Abmart, China) at room temperature for 1 h. DAPI (1:1000, C0060, Solarbio, China) was used to label cell nuclei. Images were captured using an inverted microscope (IX71, Olympus, Japan).

Appendix S4. miRNA microarray analysis

Human miRNA microarrays from Agilent Technologies (8*60 K) containing probes for 2549 human miRNAs from the miRbase V21.0 database were used. Total RNA (100 ng) extracted from bAVM or HUVEC exosome samples was used as input for sample labeling and hybridization according to the manufacturer's protocol (Agilent Technologies, USA). The microarray images were converted into spot intensity values using Scanner Control Software Rev. 7.0 (Agilent Technologies, USA). The raw data were normalized by the quantile algorithm included in the R package AgiMicroRna (López-Romero, P. BMC Genomics. 2011). Microarray experiments were performed by following the protocol of Agilent Technologies Inc. at Shanghai Biotechnology Corporation.

Supplementary Table

Table S1. Mutation allele frequency of bAVM ECs

Patient	G12D_D0	G12D_D5
AVM1	23%	8%
AVM2	14%	16%
AVM3	9%	7%
AVM4	3%	NA
AVM5	39%	NA
AVM6	41%	NA

The bAVM EC samples were numbered AVM1-AVM6. The first column represents the study sample. The next two columns represent the mutation frequencies of $KRAS^{G12D}$ at two different time points. NA: not available. bAVM - brain arteriovenous malformation, EC-endothelial cell.

Supplementary Figures

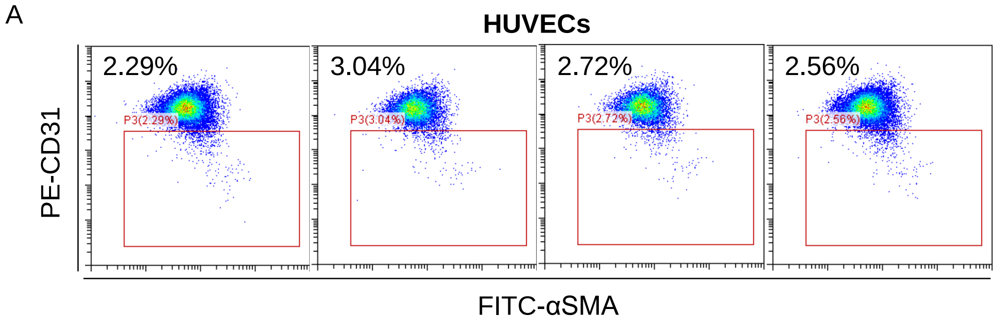

Fig S1. Flow cytometric analysis of the expression of CD31 and αSMA in HUVECs. A, Four additional flow cytometric results in HUVECs. The percentage of EndMT ECs were shown. HUVEC, human umbilical vein endothelial cell; EndMT, endothelial–mesenchymal transition.

A

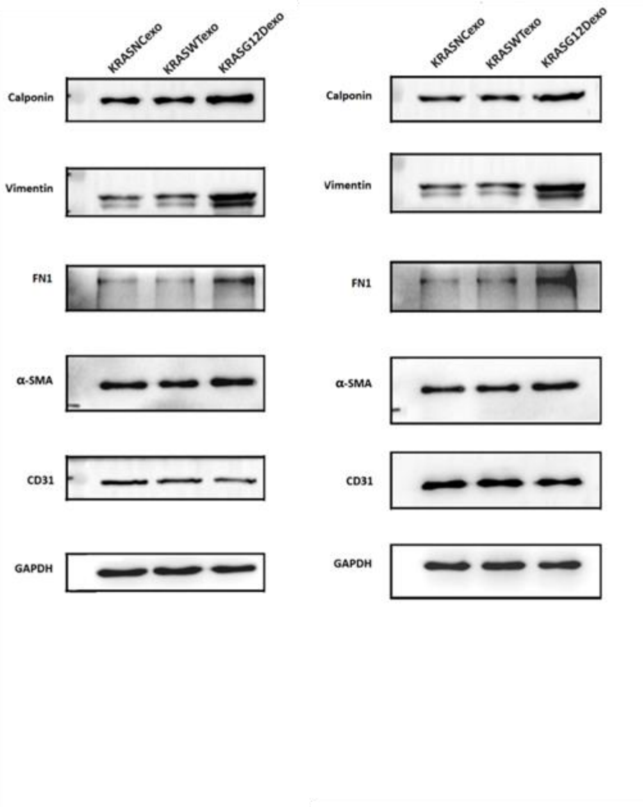

Fig S2. A, Two additional western blot of effects of $HUVEC^{G12Dexos}$, $HUVEC^{WTexos}$ or $HUVEC^{NCexos}$ on EndMT markers at protein level.

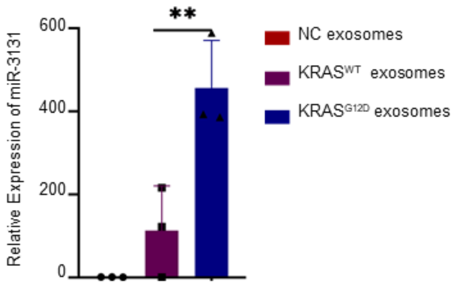

Fig S3. Bar graph of the relative expression of miR-3131 in exosomes derived from HUVECs transfected with KRAS^{G12D}, KRAS^{WT} or NC lentiviruses using RT–PCR. *P<0.05

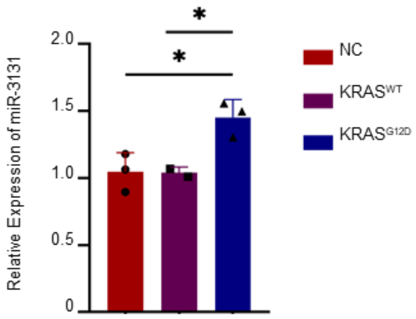

Fig S4. Bar graph of the relative expression of miR-3131 in HUVECs transfected with $KRAS^{G12D}$, $KRAS^{WT}$ or NC lentiviruses using RT–PCR. **P<0.01

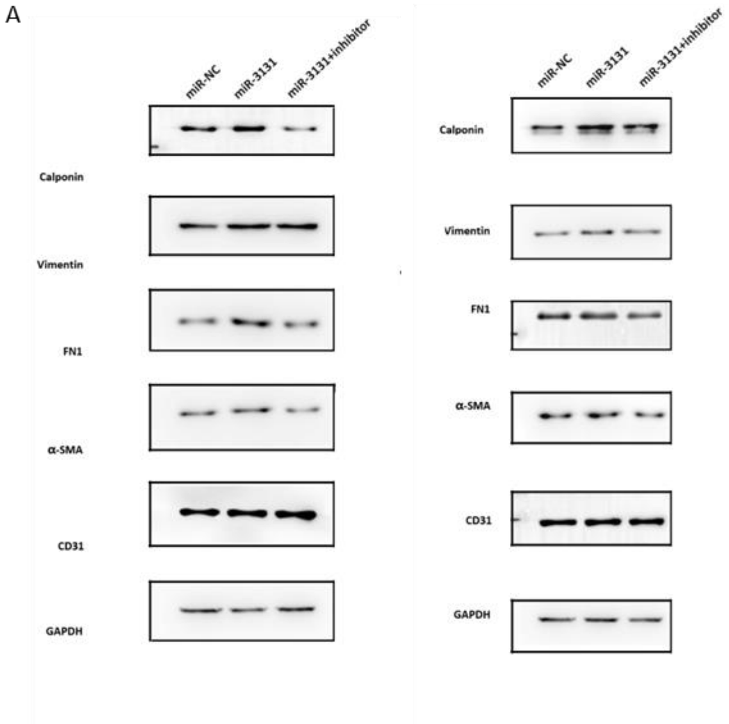

Fig S5. A, Two additional blot of effects of miR-3131 mimic and miR-3131 inhibitor on EndMT markers at protein level.

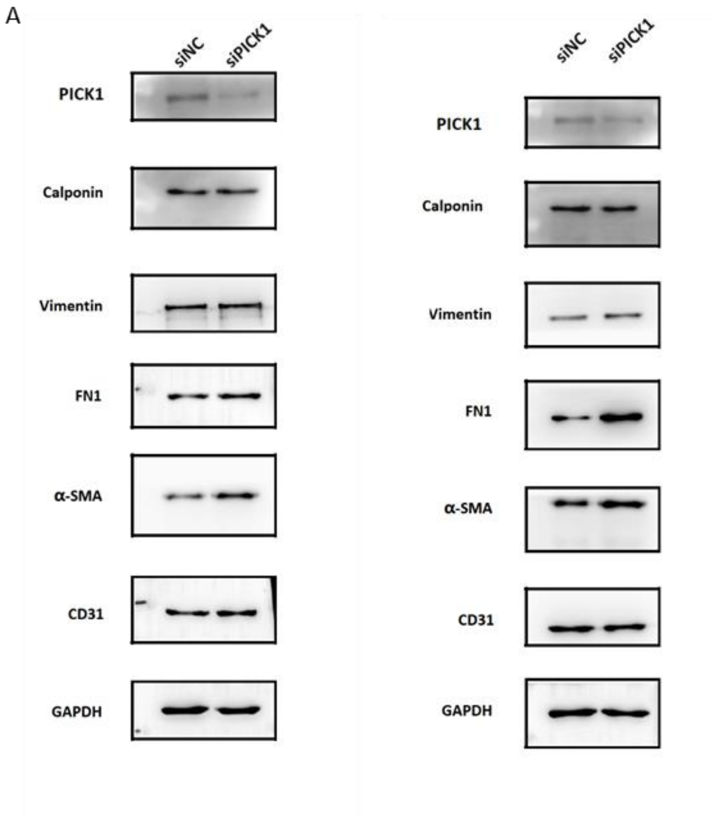

Fig S6. Two additional western blot of effects of PICK1 knockdown in HUVECs on EndMT markers at protein level.

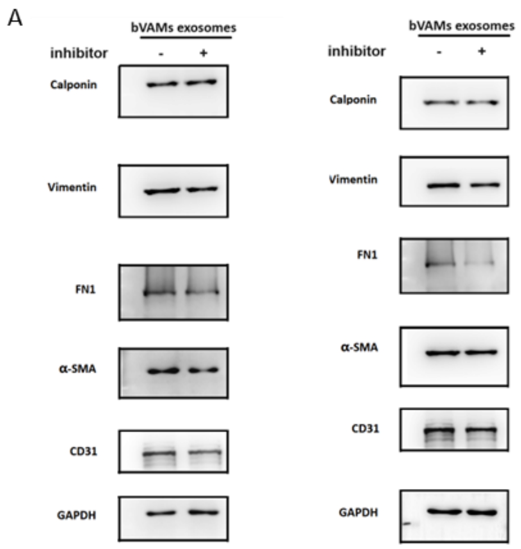

Fig S7. Two additional western blot of effects of miR-3131 inhibitor on HUVECs treated with exosomes derived from bAVM ECs on EndMT markers at protein level.

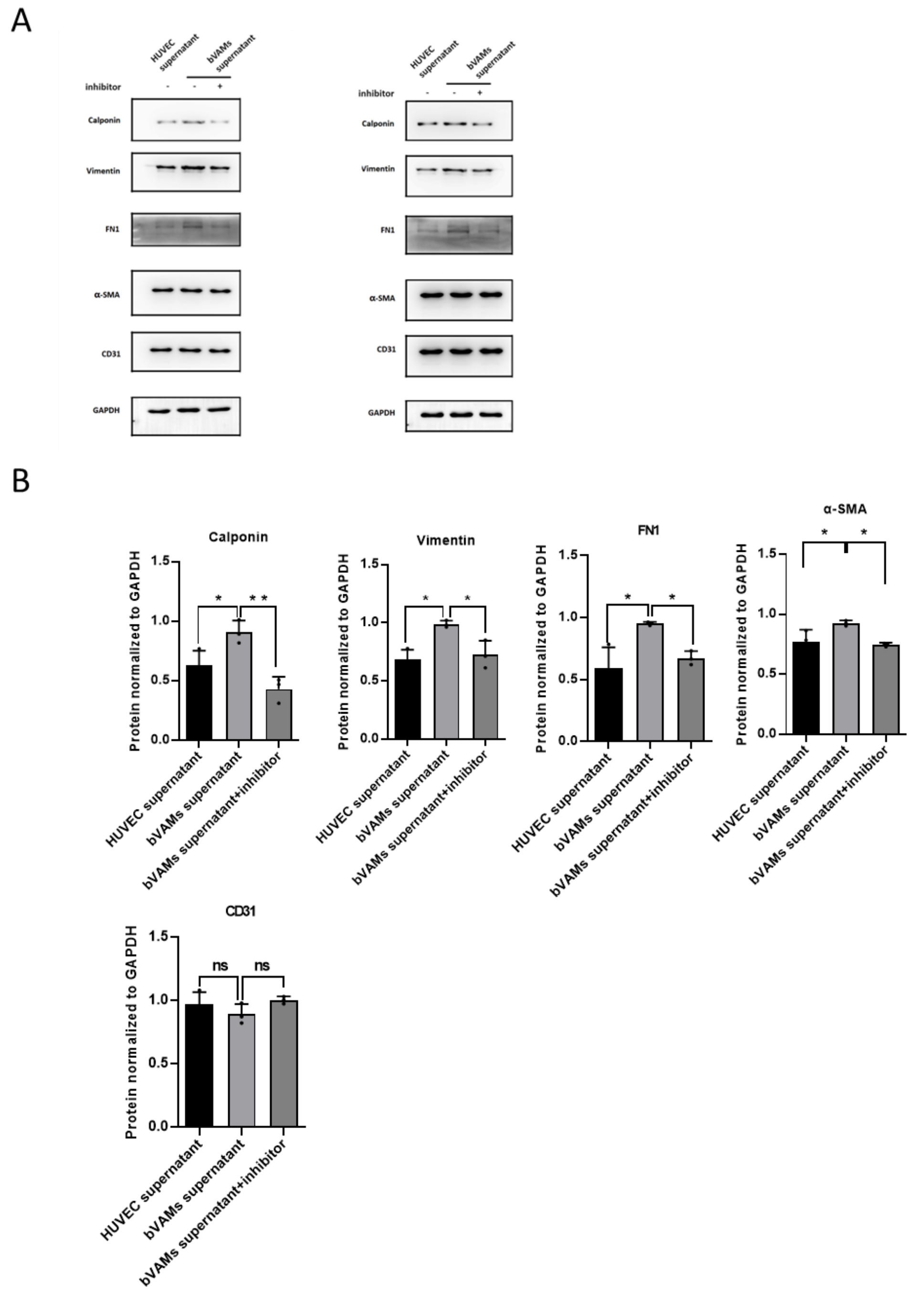

Fig S8. Effects of miR-3131 inhibitor on HUVECs treated with supernatant derived from bAVM ECs on EndMT markers at protein level. A, Two additional western blot. B, Quantification showing increase in mesenchymal markers (Calponin, Vimentin, Fibronectin, and aSMA) after treated with supernatant and reversed after transfected with miR3131 inhibitor. *: $p<0.05$, **: $p<0.01$.

A

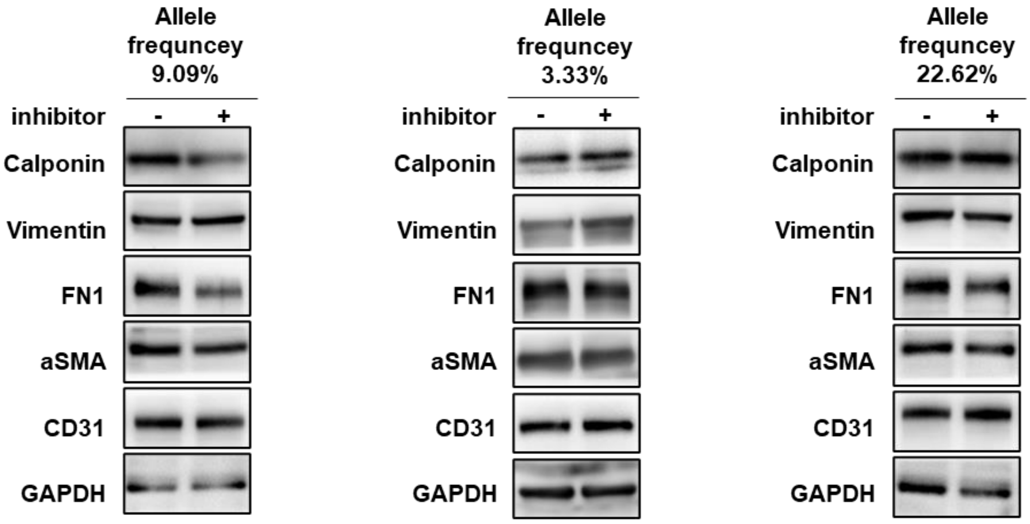

Fig S9. Western blot assay for the effect of a miR-3131 inhibitor on bAVM ECs. A, miR-3131 inhibitor on bAVM ECs with allele frequency of 9.09%, 3.33% and 22.62%. bAVM- brain arteriovenous malformation, EC-endothelial cell.

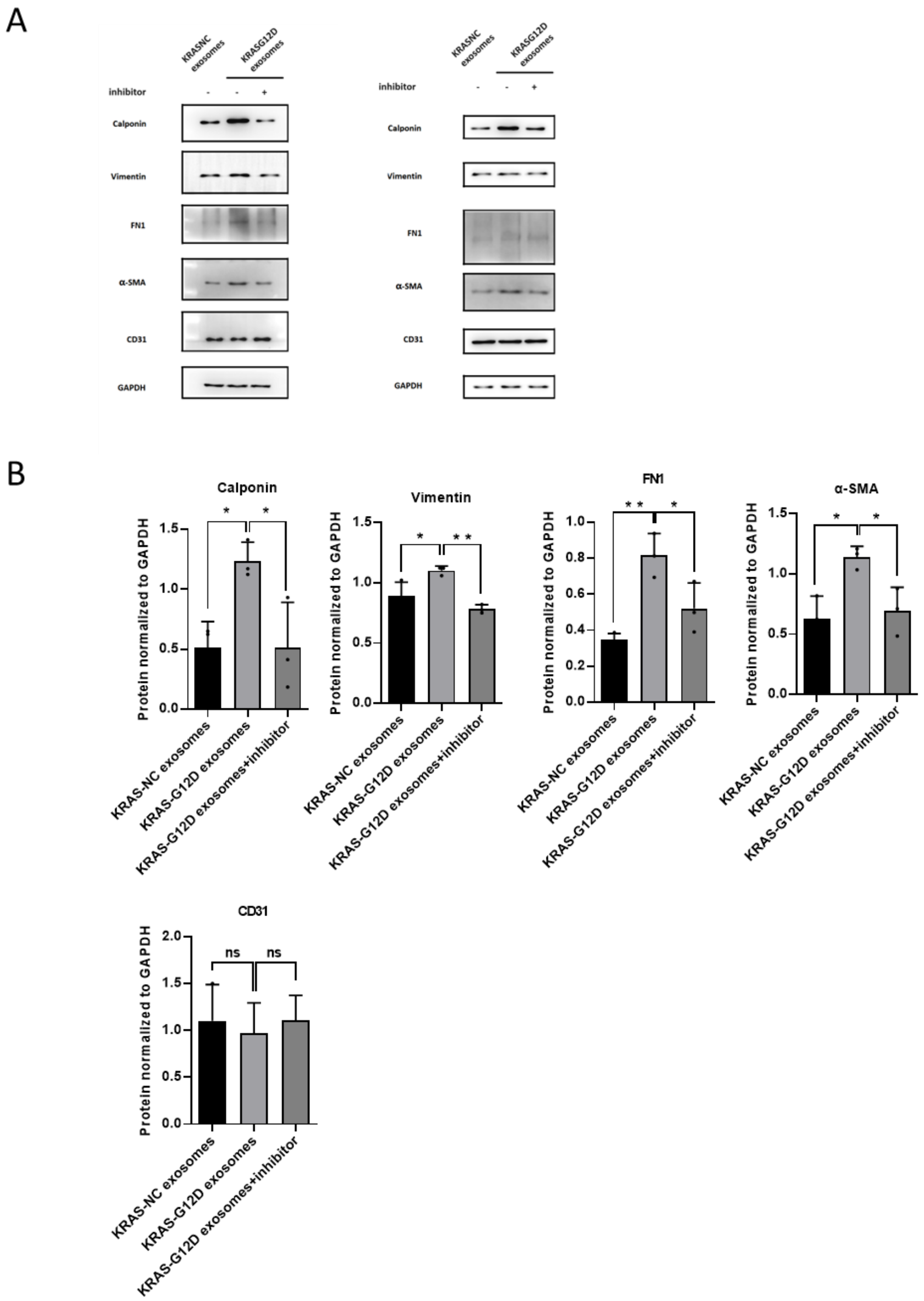

Fig S10. Effects of miR-3131 inhibitor on HUVECs treated with exosomes derived from KRAS^{G12D-} or KRAS^{NC-}transfected HUVECs on EndMT markers at protein level. A, Two additional western blot. B, Quantification showing increase in mesenchymal markers (Calponin, Vimentin, Fibronectin, and aSMA) after treated. *: p<0.05, **: p<0.01.

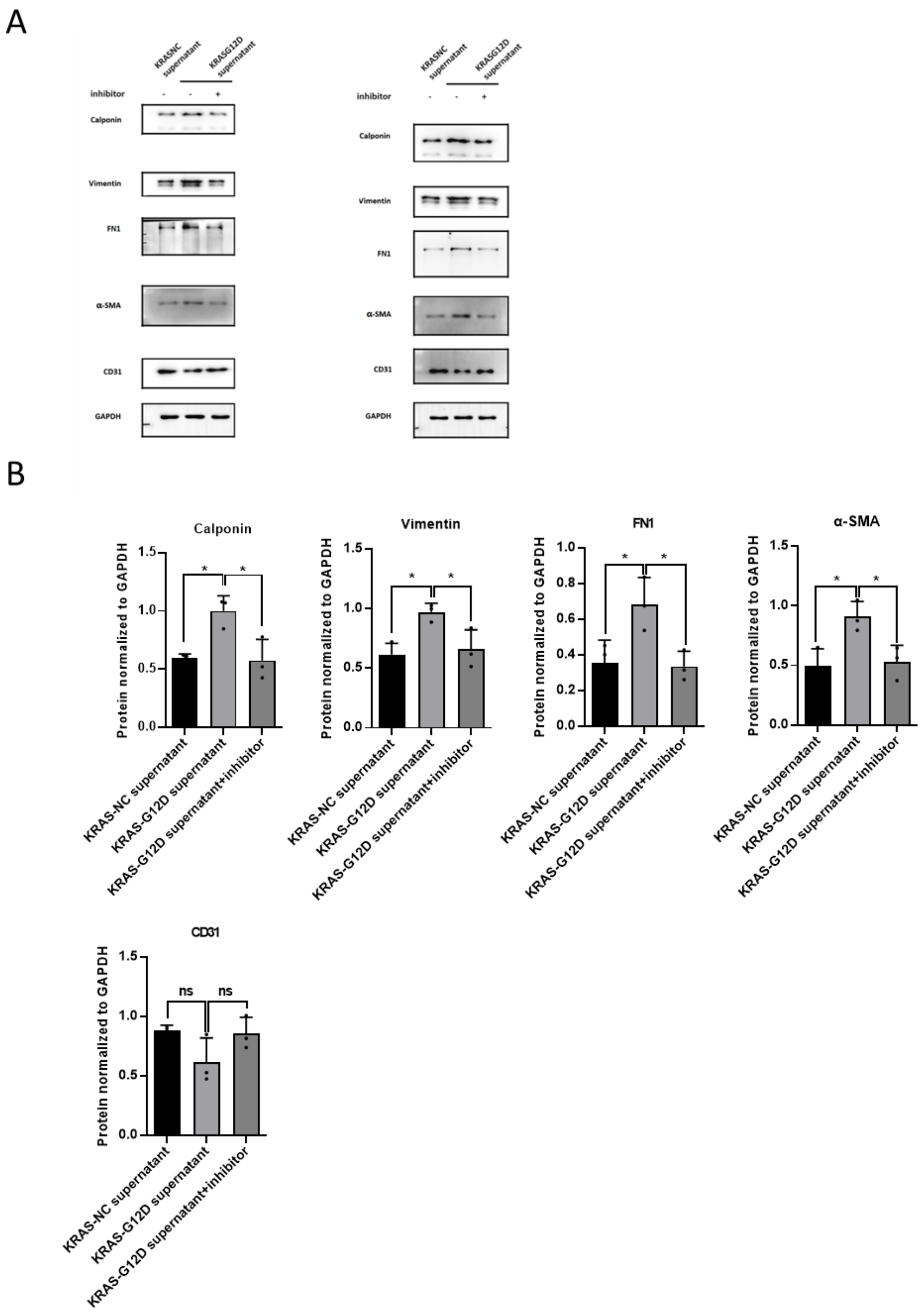

Fig S11. Effects of miR-3131 inhibitor on HUVECs treated with supernatant derived from $KRAS^{G12D-}$ or $KRAS^{NC-}$transfected HUVECs on EndMT markers at protein level. A, Two additional western blot. B, Quantification showing increase in mesenchymal markers (Calponin, Vimentin, Fibronectin, and aSMA) after treated. *: p<0.05, **: p<0.01.
